# Dok2 mediates the CD200Fc attenuation of Aβ-induced changes in glia

**DOI:** 10.1186/1742-2094-9-107

**Published:** 2012-05-29

**Authors:** Anthony Lyons, Eric J Downer, Derek A Costello, Niamh Murphy, Marina A Lynch

**Affiliations:** 1 Physiology Department, Trinity College Institute for Neuroscience, Trinity College, Dublin 2, Ireland; 2 St Matthew’s University, School of Medicine, Grand Cayman, British West Indies

**Keywords:** Aβ, CD200, Cytokines, Microglia, siRNA, Dok2, Phagocytosis

## Abstract

**Background:**

The interaction between the membrane glycoprotein, CD200 and its cognate receptor CD200 receptor (CD200R), has been shown to play a role in maintaining microglia in a quiescent state. There is evidence of increased activation under resting and stimulated conditions in microglia prepared from CD200-deficient mice compared with wild-type mice, whereas activation of the receptor by CD200 fusion protein (CD200Fc) ameliorates inflammatory changes which are evident in the central nervous system (CNS) of the mouse model of multiple sclerosis (MS), experimental autoimmune encephalomyelitis (EAE) and also in the hippocampus of aged rats. Additionally, an inverse relationship between microglial activation and expression of CD200 has been observed in animals treated with lipopolysaccharide (LPS) or amyloid-β (Aβ).

**Methods:**

We assessed the effect of CD200R activation by CD200Fc on Aβ-induced production of the pro-inflammatory cytokines, interleukin-1β (IL-1β) and tumor necrosis factor-α (TNFα) and the expression of microglial activation markers, CD68 and CD40 in cultured glia. The role played by downstream of tyrosine kinase 2 (Dok2) phosphorylation in mediating the effects of CD200R activation was evaluated by siRNA knockdown of Dok2. To further examine the impact of inflammatory changes on synaptic plasticity, the effect of CD200Fc on Aβ-induced impairment of long-term potentiation (LTP) in the CA1 region of hippocampal slices was also investigated.

**Results:**

We demonstrate that Aβ-induced increases in IL-1β, TNFα, CD68 and CD40 were inhibited by CD200Fc. The evidence suggests that Dok2 phosphorylation is a key factor in mediating the effect of CD200Fc, since Dok2 knockdown by siRNA abrogated its effects on microglial activation and inflammatory cytokine production. Consistent with evidence that inflammatory changes negatively impact on LTP, we show that the Aβ-induced impairment of LTP was attenuated by CD200Fc.

**Conclusions:**

The findings suggest that activation of CD200R and Dok2 is a valuable strategy for modulating microglial activation and may have therapeutic potential in neurodegenerative conditions.

## Introduction

It is accepted that neuroimmune regulatory proteins, which are expressed on many cells, interact with their receptors on microglia and play an important role in modulating the activity of these cells. One of the most-studied ligand-receptor pairs is CD200-CD200 receptor (CD200R). In common with other ligand-receptor pairs, CD200 is widely expressed, whereas receptor expression is confined primarily to cells of the myeloid lineage [1,2]. In the CNS, CD200 is expressed on neurons [3,4] and astrocytes [5,6]. Compelling evidence for a modulatory role for CD200-CD200R interaction has been obtained from the study of changes in CD200-deficient mice. Under resting conditions, the number of activated microglia is increased in the brain of these mice [7] and they exhibit exaggerated responses to inflammatory challenge from lipopolysaccharide (LPS) and amyloid-β (Aβ) [4,8], due possibly to increased relative expression of Toll-like receptors (TLR) 2 and 4 [7].

Exacerbated symptoms of disease have been reported with evidence of enhanced inflammatory changes in animal models of multiple sclerosis [9] and Parkinson’s disease [10]. A key change in these experimental conditions is increased microglial activation and, in these models as well as others, including *Toxoplasma gondii*-induced encephalitis [11], facial nerve transaction [9] and experimental autoimmune uveoretinitis [12], greater microglial activation was observed in CD200-deficient, compared with wild-type mice. Consistently, an inverse relationship between microglial activation and CD200 has been described in the brain of aged animals [4], in animals following ischaemic insult and in Aβ- [4] and LPS-treated animals [8].

Activation of CD200R by a CD200Fc has also been shown to decrease the symptoms of experimental autoimmune encephalomyelitis (EAE) and the associated activation of microglia/macrophages [13], and this has been reflected in its ability to decrease the inflammatory changes observed in collagen-induced arthritis [14,15]. Similarly we have recently shown that intracerebroventricular injection of CD200Fc decreased expression of markers of microglial activation in the hippocampus of aged rats [5].

The 67 amino acid cytoplasmic tail of CD200R contains an NPXY signaling motif with three tyrosine residues; the interaction between CD200 and CD200R induces phosphorylation of these residues initiating a signaling cascade [16]. The adaptor proteins, Dok1 and Dok2, are recruited to the complex leading to activation of RasGAP and SH2-containing inositol phosphatase (SHIP) [17]. At least in the case of U937 cells, it appears that the modulatory effect of CD200R activation on IL-8 production is mediated by recruitment of Dok2 and RasGAP activation [17]. Thus, knockdown of Dok2, but not Dok1, ameliorated the increase in IL-8 production following CD200R activation.

Here we assessed the effect of CD200Fc on Aβ-induced changes in glia, showing that it inhibited microglial activation and that these changes were mediated via Dok2 phosphorylation.

## Methods

### Preparation and treatment of primary glial cultures

Mixed glial cultures were prepared from one-day-old C57BL/6 mice as previously described [4]. These cultures contained approximately 70% astrocytes and 30% microglia as measured by assessing CD11b expression using flow cytometry [7].

To prepare purified microglia, cells were seeded onto 25 cm^2^ flasks and, after 24 hours, media was replaced with cDMEM containing GM-CSF (10 ng/ml) and M-CSF (20 ng/ml). After 10 days in culture, non-adherent microglia were harvested by shaking (110 rpm, two hours, room temperature), tapping and centrifuging (2,000 rpm, five minutes). The pellet was resuspended in cDMEM, the microglia were plated onto 24-well plates at a density of 1 x 10^5^ cells/ml and maintained at 37°C in a 5% CO_2_ humidified atmosphere for up to three days prior to treatment.

To prepare purified astrocytes, flasks containing the adherent astrocytes were washed with sterile PBS and 2 ml of 0.05% w/v trypsin–EDTA was added at 37°C. DMEM was then added to inhibit the trypsin, the cells were centrifuged at 2,000 *g* for three minutes and the pellet was resuspended in DMEM. Cells were plated in six-well plates at a density of 2 × 10^5^ cells/ml.

Cells were incubated in the presence or absence of Aβ_[1–40]_ (4.2 μM) plus Aβ_[1–42]_ (5.6 μM) for 24 hours. In various experiments, supernatant was taken for later analysis of cytokines, and cells were harvested for analysis by FACS, for preparation of mRNA to assess expression of markers of microglial activation or cytokines, or for preparation of cell lysate to assess expression of proteins by Western immunoblotting. In some experiments, mixed glia were pre-incubated with CD200Fc (2.5 μg/ml, R & D Systems, Minneapolis, MN, USA for 30 minutes prior to Aβ treatment.

### Analysis of cell surface markers by flow cytometry

Mixed glial cells were trypsinized (0.25% Trypsin-EDTA, Sigma, Gillingham, UK), washed three times in FACS buffer (2% FBS, 0.1% NaN_3_ in PBS) and blocked for 15 minutes at room temperature in FACS block (1:500 in FACS buffer; Mouse BD Fc Block, BD Pharmingen, Oxford, UK). Cells were incubated with APC-rat anti-mouse CD11b (1:400 in FACS buffer; BD Biosciences, Oxford, UK), FITC-rat anti-mouse CD40 (1:200 in FACS buffer; BD Biosciences, UK) and FITC-rat anti-mouse CD68 (1:200 in FACS buffer; AbD Serotec, Oxford, UK), and PE-rat anti-mouse CD200R (1:200 in FACS buffer; Abcam, Cambridge, UK). To evaluate phagocytic activity in microglia, cells were incubated in the presence of carboxylate-modified polystyrene latex beads (1:200 in FACS buffer; fluorescent yellow-green, mean particle size; 1 μm; Sigma, UK) and uptake into CD11b^+^ cells was measured. Immunofluorescence analysis was performed on a DAKO Cyan ADP 7 color flow cytometer (DAKO Cytomation, Stockport, UK {) with Summit v4.3 software (Beckman-Coulter, High Wycombe, UK).

### Analysis of expression of CD200R, CD68, CD40, IL-1β, TNFα and MIP-1a mRNA

Total RNA was extracted from harvested glial cells using a NucleoSpin® RNAII isolation kit (Macherey-Nagel Inc., Duren, Germany) and cDNA synthesis was performed on 1 μg total RNA using a High Capacity cDNA RT kit (Applied Biosystems, Darmstadt, Germany); the protocols used were according to the manufacturer’s instructions. Real-time PCR was performed as described previously [4] using an ABI Prism 7300 instrument (Applied Biosystems, Germany); the assay IDs were as follows: CD40 (Mm00441891_m1), CD68 (Mm03047341_m1), CD11b (Mm00434455_m1), IL-1β (Mm00434228_m1), TNFα (Mm00443258_m1), CD200R (Mm00491164_m1) and MIP-1α (Mm00442346_m1). Samples were assayed in duplicate and gene expression was calculated relative to the endogenous control samples (β-actin) to give an RQ value (2^− DDCt^, where CT is the threshold cycle).

### Assessment of supernatant concentrations of IL-1β and TNFα

IL-1β and TNFα were assessed by ELISA as previously described [4] (Duoset, R & D Systems, Minneapolis, MN, USA). Briefly, standards or supernatant samples (100 μl) were added to antibody-coated 96-well plates and incubated for two hours at room temperature, plates were washed and samples were incubated in detection antibody for two hours. Plates were washed and incubated in horseradish peroxidase-conjugated streptavidin (1:200 in PBS containing 1% BSA) for 20 minutes at room temperature. Substrate solution (tetramethylbenzidine, Sigma, UK) was added, incubation continued at room temperature in the dark for 30 minutes and the reaction was stopped using 1 M H_2_SO_4_. Absorbance was read at 450 nm, values were corrected for protein and expressed as pg/ml protein.

### Assessment of CD200 expression and Dok2 phosphorylation by Western immunoblotting

Aβ-treated microglia were harvested in lysis buffer (50 ml; composition in mM: Tris–HCl 10, NaCl 50, Na_4_P_2_O_7_.H_2_O 10, NaF 50, 1% Igepal, phosphatase inhibitor cocktail II and III, protease inhibitor cocktail; all Sigma, UK) and stored at −20°C. For analysis, samples were added to 4x SDS sample buffer (composition: Tris–HCl 100 mM, pH 6.8, 4% SDS, 2% bromophenol blue, 20% glycerol; all Sigma, UK) and heated to 95°C for five minutes. Samples were separated on 4 to 20% Bis-Tris gels (Invitrogen, Paisley, UK). Proteins were transferred to nitrocellulose membrane (Schleicher and Schuell, Dassel, Germany) and blocked for one hour in Tris-buffered-saline-0.05% Tween 20 (TBS-T) and 5% non-fat dried milk/TBS-T at room temperature. Membranes were incubated overnight at 4°C with anti-Phospho-Dok2 (1:1,000; R & D Systems, USA) or b-actin (1:5,000; Sigma, UK) in 2% non-fat dried milk/TBS-T, washed, and incubated with a horseradish peroxidase (HRP) conjugated anti-goat antibody (1:5,000; Jackson Immunoresearch, West Grove, PA, USA) in 2% non-fat dried milk/TBS-T for 1.5 h.

Aβ-treated astrocytes were harvested as described and CD200 expression assessed by probing membranes with anti-CD200 antibody (1:2,000; R & D Systems, USA) in 2% non-fat dried milk/TBS-T, washed, and incubated with a HRP conjugated anti-goat antibody (1:5,000; Jackson Immunoresearch, West Grove, PA, USA) in 2% non-fat dried milk/TBS-T for 1.5 h.

Immunoreactive bands were detected using Immobilon Western chemiluminescent substrate (Millipore, Billerica, MA, USA) and images captured using the Fujifilm LAS-4000 imager imager (Brennan and Co., Dublin, Ireland). To quantify expression of the proteins in some cases, densitometric analysis was carried out using ImageJ (http://rsbweb.nih.gov/ij/) (National InstituteS of Health, Bethesda, MD, USA) . Values are presented as mean ± S.E.M., normalized to β-actin.

### RNA interference

Custom ON-TARGET plus smart pool small interfering RNA (siRNA) targeting mouse Dok2 (Gen bank^TM^ accession number NM_010071) was purchased from Dharmacon (Lafayette, CO, USA). Primary microglial cells were transfected with Dok2 siRNA (5 nM) using DharmaFECT 1 transfection reagent (Dharmacon). After 72 hours (52% Dok2 knockdown), cells were pre-treated with CD200Fc prior to Aβ treatment. A control siRNA duplex containing at least four mismatches of any mouse gene (ON-TARGET plus Non-targeting siRNA) was used in parallel experiments. Effective Dok2 knockdown was determined using confocal microscopy and Western blotting.

### Analysis of LTP in hippocampal slices

Hippocampal slices (400 μm), were prepared from male C57BL/six mice (five to seven months old), using a McIlwain tissue chopper (Mickle Laboratory, Surrey, UK). Slices were maintained in oxygenated artificial cerebrospinal fluid (aCSF; composition in mM: 125 NaCl, 1.25 KCl, 1 CaCl_2_, 1.5 MgCl_2_, 1.25 KH_2_PO_4_, 25 NaHCO_3_, and 10 d-glucose) at room temperature (21 to 23°C) in a holding chamber for a minimum of one hour before being transferred to a submersion recording chamber, where they were perfused with oxygenated aCSF (containing 2 mM CaCl_2_; 2–3 ml/min; 21 to 23°C). The Schaffer collateral-commissural pathway was stimulated at 0.033 Hz (0.1 ms duration; approximately 50% of maximal EPSP amplitude) using a bipolar tungsten stimulation electrode (Advent Materials, Oxford, UK). Field excitatory postsynaptic potentials (EPSPs) were recorded from the CA1 stratum radiatum using a monopolar glass recording electrode filled with aCSF. Stable baseline EPSPs were recorded for 15 to 20 minutes prior to application of theta-burst stimulation (TBS; 10 trains (4 pulses at 100 Hz) repeated at 5 Hz) or pharmacological agents. Aβ_[1–40]_ (500 nM) was applied to the perfusate 40 minutes prior to delivery of TBS. In a separate group of experiments, slices were perfused with CD200Fc (2 mg/ml) for 20 minutes prior to application of Aβ. As an additional control, slices were treated with mouse IgG (2 mg/ml; Sigma, UK) for 20 minutes prior to Aβ application. Data were acquired using WinWCP v4.0.7 software (Dr. J. Dempster, Strathclyde, UK) and evoked EPSPs were normalised to the mean EPSP slope recorded in the five-minute period prior to LTP induction. LTP was measured as a mean value of the final five minutes of recording (55 to 60 minutes post-TBS).

### Statistical analysis

Where appropriate, data were analyzed using Student’s *t*-test for independent means. In most cases, data were evaluated using analysis of variance (ANOVA) followed by *post hoc* Student Newman–Keuls test to determine which conditions were significantly different from each other. Data are expressed as means with standard errors.

## Results

### Aβ modulates CD200R-associated signaling

**Figure 1:**
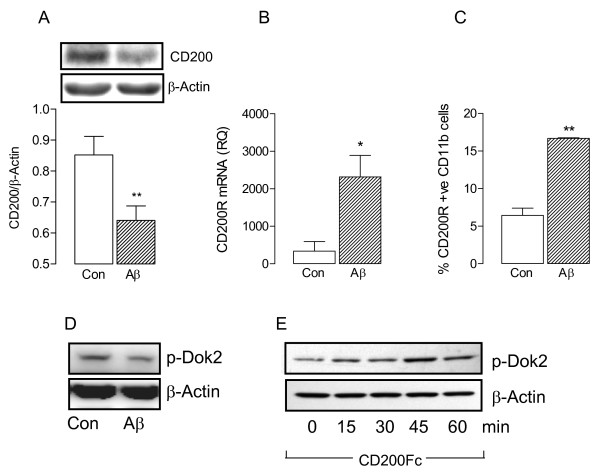
**Aβ modulates CD200R-associated signaling**. **A**. Incubation of purified astrocytes in the presence of Aβ decreased CD200 expression as illustrated in the sample immunoblot and in the densitometric data (**P < 0.01; Student’s *t*-test for independent means; n = 6). **B** and **C**. Aβ significantly increased CD200R mRNA on purified microglia (**B**) and the percentage of CD200R^+^ CD11b^+^ cells (**C**; *P < 0.05; **P < 0.01; Student’s *t*-test for independent means; n = 6). **D** and **E**. Whereas Aβ decreased Dok2 phosphorylation (**D**), CD200Fc increased its phosphorylation in a time-dependent manner (**E**).

CD200 is expressed on many cells, including neurons and astrocytes [3,5], whereas CD200R is expressed primarily on cells of the myeloid lineage, including microglia [4,9]. Here we report that incubation of astrocytes in the presence of Aβ decreased CD200 as shown by Western blot (**P < 0.01; Student’s *t*-test; Figure 1A). Incubation of microglia in the presence of Aβ increased CD200R mRNA (*P < 0.05; Student’s *t*-test; Figure 1B) and the number of CD11b^+^ cells that expressed CD200R (**P < 0.01; Student’s *t*-test; Figure 1C). Activation of CD200R plays a role in maintaining microglia in a quiescent state [4] and the evidence suggests that, at least in macrophages [17,18], this relies on activation of Dok2. Whereas Aβ decreased phosphorylation of Dok2 in mixed glial cells (Figure 1D), CD200Fc increased phosphorylation of Dok2 in a time-dependent manner (Figure 1E).

### CD200Fc attenuated the Aβ-induced increases in markers of microglial activation and phagocytosis

**Figure 2:**
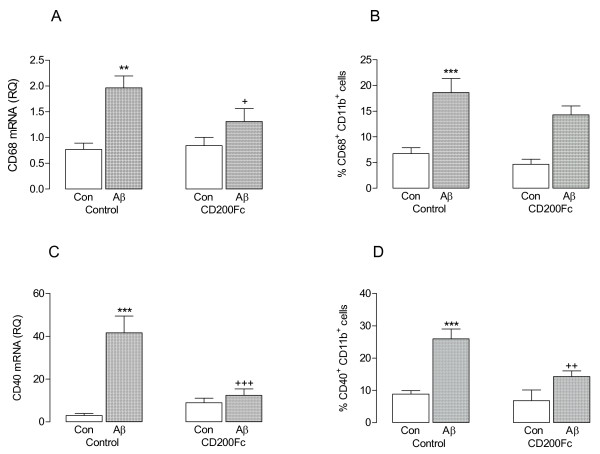
**CD200Fc attenuated the Aβ-induced increases in markers of microglial activation.** Incubation of mixed glia in the presence of Aβ significantly increased expression of CD68 mRNA (**A**) and CD40 mRNA (**C**; **P < 0.01; ***P < 0.001; ANOVA; n = 6). Aβ also increased the percentage of CD11b^+^ cells which expressed CD68 (**B**) and CD40 (**D**; ***P < 0.001; ANOVA; n = 6). Addition of CD200Fc to the incubation medium significantly attenuated the Aβ-induced changes in CD68 mRNA, CD40 mRNA and the percentage of CD11b^+^ cells which expressed CD40 (^+^*P* < 0.05; ^++^*P* < 0.01; ^+++^*P* < 0.001).

Aβ significantly increased CD68 mRNA (**P < 0.01; ANOVA; Figure 2A) and this effect was attenuated in Aβ-treated cells incubated with CD200Fc (^+^*P* < 0.05; ANOVA). To assess whether the change in gene expression translated into a change in expression of CD68 on microglia, cells were evaluated using FACS and the data indicated that Aβ significantly increased the number of CD11b^+^ cells which stained positively for CD68 (**P < 0.01; ANOVA; Figure 2B). The Aβ-induced effect was attenuated by CD200Fc but the effect did not reach statistical significance. CD40, another marker of microglial activation, was similarly modulated by Aβ and CD200Fc; thus Aβ significantly increased CD40 mRNA and the number of CD11b^+^ cells which stained positively for CD40 (***P < 0.001; ANOVA), while CD200Fc significantly attenuated the Aβ-induced effects (^++^*P* < 0.01; ^+++^*P* < 0.001; ANOVA; Figure 2C, D).

**Figure 3:**
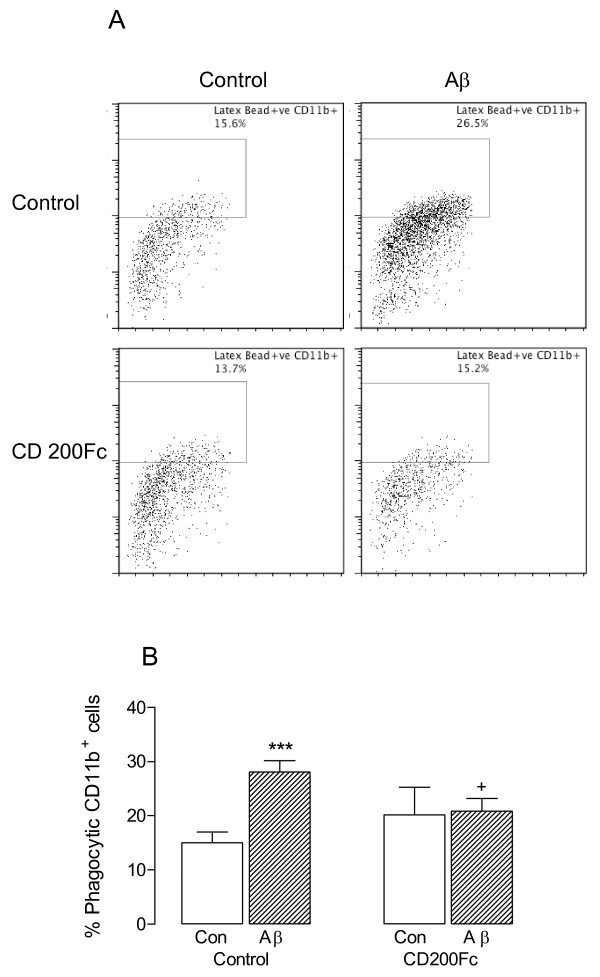
**CD200Fc attenuated the Aβ-induced increase in phagocytosis**. **A**. Incubation of mixed glia in the presence of Aβ significantly increased the number of cells that internalized fluorescently-labeled latex beads (compare top two panels) and, although CD200Fc alone had no impact on latex bead internalization (bottom left-hand panel), CD200Fc attenuated the Aβ-induced change (compare right-hand panels). **B**. Mean data indicated a significant effect of Aβ on internalization of fluorescently-labelled latex beads (***P < 0.001; ANOVA; n = 6) which was significantly attenuated by CD200Fc (^+^*P* < 0.05; ANOVA).

Microglia can adopt many activated phenotypes, one of which is a phagocytic phenotype and the present data indicate that, as well as altering expression of cell surface markers of activation, Aβ also increases phagocytic activity. Phagocytosis, assessed by evaluating engulfment of fluorescently-labeled latex beads by CD11b^+^ cells, was significantly increased by Aβ (***P < 0.001; ANOVA; Figure 3); CD200Fc ameliorated the Aβ-induced effect (^+^*P* < 0.05; ANOVA) suggesting that the signaling events triggered by CD200R activation modulate microglial phagocytic function.

**Figure 4:**
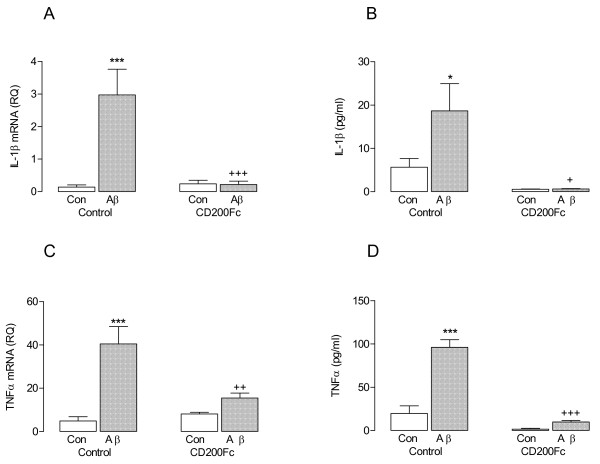
**CD200Fc attenuated the Aβ-induced increases in IL-1β and TNFα**. Incubation of mixed glia in the presence of Aβ significantly increased expression of IL-1β mRNA (**A**) and TNFα mRNA (**C**; ***P < 0.001; ANOVA; n = 6). Aβ also increased supernatant concentrations of IL-1β (**B**) and TNFα (**D**; *P < 0.05; ***P < 0.001; ANOVA; n = 6). Addition of CD200Fc to the incubation medium significantly attenuated the Aβ-induced changes in these four parameters (^+^*P* < 0.05; ^++^*P* < 0.01; ^+++^*P* < 0.001).

Activated microglia also release inflammatory cytokines and, in parallel with the changes in markers of microglial activation and phagocytic function, Aβ significantly increased IL-1β and TNFα mRNA expression in cells (***P < 0.001; ANOVA; Figure 4A, C) and release of both cytokines from mixed glia (*P < 0.05; ***P < 0.001; ANOVA; Figure 4B, D). The data indicate that CD200Fc significantly attenuated these Aβ-induced changes (^+^*P* < 0.05; ^++^*P* < 0.01; ^+++^*P* < 0.001; ANOVA).

### Dok2 mediated the effects of CD200Fc

**Figure 5:**
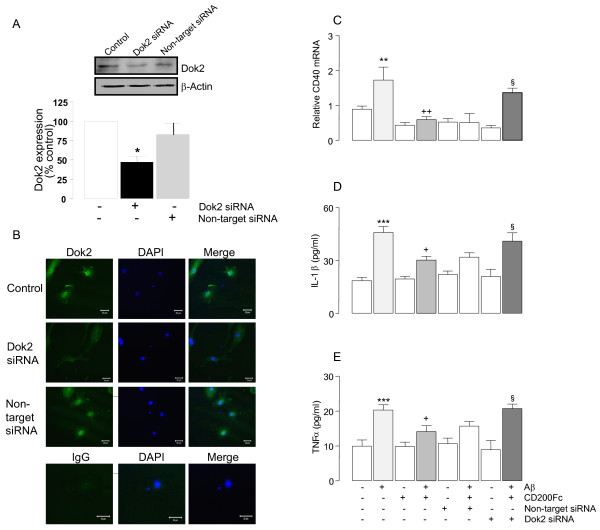
**Dok2 mediated the effects of CD200Fc**. Efficiency of Dok2 siRNA knock-down was measured by western blot (**A**) and immunofluoresence (**B**). Incubation of microglia in the presence of Aβ significantly increased expression of CD40 mRNA (**C**) and release of IL-1β (**D**) and TNFα (**E**; **P < 0.01; ***P < 0.001; ANOVA; n = 6). Addition of CD200Fc to the incubation medium significantly attenuated these Aβ-induced changes (^+^*P* < 0.05; ^++^*P* < 0.01). Whereas non-target siRNA exerted no significant effect, Dok2 siRNA prevented the action of CD200Fc so that there was a significant difference in CD40 mRNA and in supernatant concentrations of IL-1β and TNFα prepared from cells treated with Aβ + CD200Fc compared with those treated with Aβ + CD200Fc + Dok2 siRNA (^§^*P* < 0.05; ANOVA).

The mechanism by which CD200Fc modulates Aβ-induced changes is unclear but activation of CD200R results in phosphorylation of Dok2 and the evidence suggests that this is pivotal for the negative effects of CD200 on microglial function [17,18]. To evaluate whether these signaling events underlie the modulatory effect of CD200Fc on Aβ, the effect of *dok2* siRNA was assessed. The efficiency of Dok2 knockdown was assessed by Western blot (Figure 5A) and immunofluoresence (Figure 5B). The quantitative data obtained from Western immunoblotting indicated that there was 52% reduction in Dok2 expression relative to a non-targeted control siRNA (^*^P < 0.05; ANOVA; Figure 5A), whereas immunofluorescent images suggested that the reduction was greater. The effect of Dok2 knock-down was assessed on expression of CD40 mRNA and on cytokine release. Aβ significantly increased CD40 mRNA (**P < 0.01; ANOVA; Figure 5C) and this effect was attenuated by including CD200Fc in the incubation medium (^++^*P* < 0.01; ANOVA), mirroring the data presented in Figure 2. However, this modulating effect of CD200Fc was partially attenuated in Dok2 siRNA-treated cells; thus there was a significant difference in CD40 mRNA expression in cells which were incubated in Aβ + CD200Fc compared with those incubated in the presence of Aβ + CD200Fc + Dok2 siRNA (^§^*P* < 0.05; ANOVA; Figure 5C). We also assessed supernatant concentrations of IL-1β and TNFα and the results show a similar pattern. Incubation of cells in the presence of Aβ significantly increased IL-1β and TNFα (***P < 0.001; ANOVA; Figure 5D, E) and this effect was attenuated by CD200Fc (^+^*P* < 0.05; ANOVA). Pretreatment of cells with Dok2 siRNA prevented the modulating effect of CD200Fc so that there was a significant difference in cytokine concentration in supernatant prepared from cells treated with Aβ + CD200Fc compared with those treated with Aβ + CD200Fc + Dok2 siRNA (^§^*P* < 0.05; ANOVA).

### CD200Fc relieves the Aβ-mediated deficit in LTP

**Figure 6:**
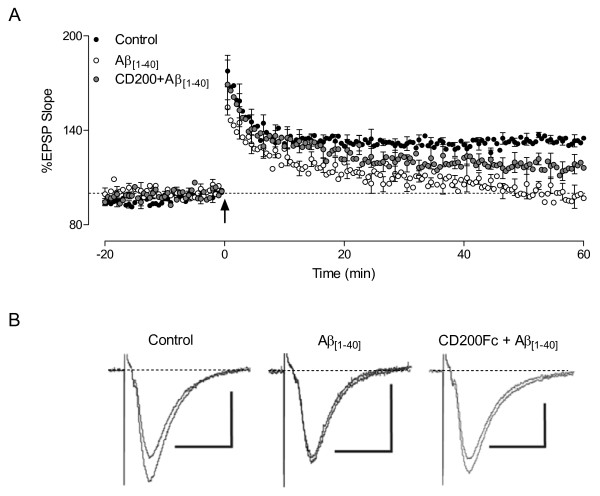
**CD200Fc relieves the Aβ-mediated deficit in LTP**. **A.** Perfusion of Aβ_[1–40]_ (500 nM) for 40 minutes prior to TBS (arrow) significantly impaired LTP in area CA1 of hippocampal slices (n = 6) compared with controls (n = 5; *P* < 0.001). Pre-treatment with CD200Fc (2 mg/ml) for 20 minutes prior to application of Aβ_[1–40]_ significantly increased the level of LTP compared with values obtained in the presence of Aβ alone (n = 4; *P* < 0.05; ANOVA). **B**. Sample EPSP traces (average of four consecutive recordings; scale bar: 1 mV/20 ms), taken immediately prior to application of TBS and 60 minutes post-LTP induction, in control and Aβ-treated slices, and from a slice co-treated with CD200Fc and Aβ.

It is well established that inflammatory changes negatively impact on LTP [19] and also that LTP is inhibited by Aβ [20]. We considered that inhibiting the Aβ-induced inflammatory changes by CD200Fc may result in restoration of LTP. Here we confirm that LTP in hippocampal slices was significantly impaired in response to application of Aβ (***P < 0.001; ANOVA) and show that this deficit was significantly attenuated when Aβ was applied in the presence of CD200Fc (*P < 0.05; ANOVA; Figure 6). The detrimental effects of Aβ on LTP were not alleviated in the presence of a control mouse IgG (data not shown).

## Discussion

The CNS is protected from insult by several mechanisms and one of these is the control of microglial activation by neuroimmune regulatory molecules. The significant finding of this study is that CD200Fc ameliorates the Aβ-induced microglial activation and production of inflammatory cytokines, and that this is dependent on Dok2 phosphorylation.

The absence of CD200 is associated with increased inflammation and symptoms in EAE and collagen-induced arthritis [9] and the evidence indicates that CD200Fc ameliorates these changes [13-15,21]. CD200Fc has also been shown to inhibit mast cell degranulation [22], to attenuate IFNγ- and IL-17-induced cytokine secretion from peritoneal cells [23] and to decrease LPS-induced activation of microglia prepared from spinal cord [13]. The present findings reveal that CD200Fc modulates Aβ-induced microglial activation and production of cytokines from these cells. Significantly, this is coupled with the ability of CD200Fc to attenuate the Aβ-induced inhibition of LTP. The importance of this finding is that it may offer a strategy to decrease microglial activation, and the consequences of the associated inflammatory changes, which appears to contribute to the pathogenesis of Alzheimer’s disease (AD).

The mechanism underlying CD200R-mediated signaling is distinct from most inhibitory receptors which contain immunoreceptor tyrosine-based inhibitory motifs (ITIM) and consequently signal through recruitment of Src homology 2 domain containing phosphatases (SHP). Instead, CD200R-triggered signaling relies on recruitment and subsequent phosphorylation of Dok [17]. The present data indicate that CD200Fc triggers Dok2 activation in microglia and we, therefore, investigated the possibility that knocking down Dok2 might prevent the ability of CD200Fc to modulate Aβ-induced changes. The data confirmed that CD200Fc attenuated the Aβ-induced up-regulation of CD40 mRNA expression and the release of IL-1β and TNFα from glia and importantly demonstrated that the effect of CD200Fc was blocked in cells that were incubated in the presence of Dok2 siRNA. We can conclude that in microglia the CD200R-mediated modulatory effects are dependent on Dok2 phosphorylation. However, Dok1 and Dok2 are both negative regulators of LPS-induced inflammation and previous evidence has indicated that stimulating macrophages prepared from Dok1^−/−^ or Dok2^−/−^ mice with LPS resulted in a greater production of nitric oxide, increased the number of TNFα-producing cells and markedly increased ERK phosphorylation [24]. While CD200R engagement triggers phosphorylation of both adaptor proteins [17,18], recent evidence indicates that activation and recruitment of Dok2 and the subsequent activation of RasGAP are the key events in CD200R-induced immune regulatory function in myeloid cells [17]; the present data are broadly consistent with these findings. Interestingly, kinetic analysis using U937 macrophages suggests that Dok1 negatively regulates Dok2 with evidence indicating that when Dok1 is knocked down, phosphorylation of Dok2 is increased and recruitment of RasGAP is enhanced [18].

The ability of Aβ to induce microglial activation is well-documented [25-27] and indeed this action of Aβ, and the ensuing inflammatory changes, has been proposed as one mechanism which may contribute to the pathogenesis of Alzheimer’s disease [28]. However, the molecular changes that trigger Aβ-induced microglial activation are still poorly understood, thus limiting the development of strategies which may reduce its negative effects. Previously, we have reported that the microglial activation induced by Aβ *in vivo* was accompanied by a decrease in CD200 expression on neurons [4] raising the possibility that interaction of CD200 with CD200R may modulate Aβ-induced changes. Here we show that Aβ decreases CD200 expression of astrocytes as well as neurons and that, in the context of mixed glia, the interaction between CD200 on astrocytes and CD200R on microglia is a significant factor in controlling microglial activation. We used CD200Fc to stimulate CD200R and the data show that incubating cells in its presence is capable of reducing expression of markers of microglial activation in Aβ-treated cells. Both CD40 and CD68 mRNA were increased in Aβ-treated mixed glia, and importantly, FACS analysis revealed that these changes were mirrored by increased expression of both markers on CD11b^+^ microglia. Incubating cells in the presence of CD200Fc attenuated these Aβ-induced changes. Similarly, Aβ increased the expression and release of IL-1β and TNFα as previously reported [27] and CD200Fc also attenuated these changes.

In addition to their ability to up-regulate cell surface markers of activation and inflammatory cytokines and chemokines, activated microglia may also exhibit increased phagocytosis. In this study, phagocytosis of fluorescently-labelled latex beads by CD11b^+^ cells was increased by Aβ. This finding is similar to previous observations in which Aβ was shown to increase phagocytosis of fluorescent microspheres, albeit in BV2 cells rather than primary glial cells [29,30]. In parallel with its ability to exert an inhibitory effect on Aβ-induced changes, CD200Fc also blocked the Aβ-induced phagocytosis. The present evidence, therefore, suggests that CD200Fc reverses several aspects of microglial function, specifically their secretory and phagocytic ability.

Recent data from this laboratory have shown that intracerebroventricular injection of CD200Fc attenuated the age-related and LPS-induced deficit in LTP in the dentate gyrus of urethane-anaesthetized rats *in vivo*[5]. CD200Fc decreased MHCII mRNA in both models providing further evidence that LTP is sustained when microglia are in a quiescent state [31,32]. However, the modulatory effects of CD200R activation on inflammatory changes, and particularly microglial/macrophage activation, are perhaps best characterized in EAE. In CD200-deficient mice, the onset of the symptoms is more rapid and the inflammatory changes, including activation of macrophages and microglia, are more pronounced [9]. In contrast, the attenuated disease observed in Wlds mice has been attributed to the increased expression of CD200 on neurons in these mice [33]. Similarly, CD200Fc has been shown to attenuate the inflammatory changes associated with EAE [13], while a blocking anti-CD200 antibody reversed the attenuated disease in Wlds mice [33] and resulted in exaggerated symptoms and a greater numbers of activated macrophages in the spinal cord of rats in which EAE was induced [34]. Recent data have indicated that a CD200R blocking antibody also enhanced microglial activation and neurodegenerative changes in a mouse model of Parkinson’s disease [35]. These preclinical data have been broadly supported by studies undertaken in clinical samples. Thus, increased expression of markers of macrophage or microglial activation and inflammatory cytokines, accompanied by decreased expression of CD200 has been observed in active and inactive lesions in the CNS obtained from postmortem tissue of individuals with multiple sclerosis [6] and a decrease in expression of CD200 has been reported in areas of the brain which exhibit pathology in Alzheimer’s disease [36].

It is consistently reported that factors which induce inflammatory changes have a negative impact on synaptic plasticity and, therefore Aβ, like LPS, inhibits LTP *in vivo* and *in vitro*[20,37], whereas factors that block the Aβ- and LPS-induced inflammatory changes, such as the anti-inflammatory cytokine, IL-4, exerts a restorative effect [38]. Here we observed that LTP is impaired in area CA1 following application of Aβ to hippocampal slices and, importantly, that when Aβ was applied to slices in the presence of CD200Fc, LTP was partially restored. This finding provides additional support for the hypothesis that the negative effect of inflammation on synaptic plasticity can be alleviated when the inflammatory changes are suppressed. However, it also reveals that modulating the Aβ-induced inflammatory changes by directly down-regulating microglial activity with CD200Fc exerts a marked effect on neuronal function.

## Conclusion

This study highlights the negative effect that activated microglia exert on synaptic plasticity but, importantly, it identifies the fact that activation of CD200R or triggering phosphorylation of Dok2 in microglia provides a potential target for attenuating the effects of Aβ on neurons.

## Abbreviations

Aβ: Amyloid-β; aCSF: Artificial cerebrospinal fluid; CD200Fc: CD200 fusion protein; CNS: Central nervous system; DMEM: Dulbecco’s modified Eagle’s medium; Dok2: Downstream of tyrosine kinase 2; EAE: Experimental autoimmune encephalomyelitis; EDTA: Ethylenediaminetetraacetic acid; ELISA: Enzyme-linked immunosorbent assay; EPSP: Excitatory post-synaptic potential; FACS: Fluorescence-activated cell sorting; GM-CSF: Granulocyte-macrophage colony-stimulating factor; IL-1β: Interleukin-1β; ITIM: Immunoreceptor tyrosine-based inhibitory motifs; LPS: Lipopolysaccharide; LTP: Long-term potentiation; M-CSF: Macrophage colony-stimulating factor; PBS: Phosphate buffered saline; PCR: Polymerase chain reaction; RasGAP: Ras GTPase activating protein; SHIP: SH2-containing inositol phosphatase; SHP: Src homology 2 domain containing phosphatase; TLR: Toll-like receptor; TNFα: Tumor necrosis factor-α; Wlds: Slow Wallerian degeneration.

## Competing interests

The authors declare that they have no competing interests.

## Authors’ contributions

AL contributed to the design of the study, performed the majority of Aβ and CD200Fc treaments, Western blot experiments, Real-Time PCR and FACS analysis, and reviewed and organized the data. DAC performed all LTP related experiments, and Western blot for CD200 in astrocytes. EJD performed all Dok2 siRNA experiments. NM contributed to the initial experiments and MAL directed the overall study, analysis of the data, and wrote and reviewed the manuscript. All authors have read, reviewed and approved the final manuscript.
